# Integrated Network Pharmacology and Experimental Verification to Explore the Molecular Mechanism of Hedysarum Multijugum Maxim–Curcumae Rhizoma Herb Pair for Treating Non-Small Cell Lung Cancer

**DOI:** 10.3389/fonc.2022.854596

**Published:** 2022-03-30

**Authors:** Shaopu Hu, Mengxue Ge, Shuixiu Zhang, Min Jiang, Kaiwen Hu, Lei Gao

**Affiliations:** ^1^ Beijing University of Chinese Medicine, Beijing, China; ^2^ Department of Oncology, Dongfang Hospital, Beijing University of Chinese Medicine, Beijing, China; ^3^ Department of Integrated Management, Dongfang Hospital, Beijing University of Chinese Medicine, Beijing, China

**Keywords:** Hedysarum Multijugum Maxim, Curcumae Rhizoma, network pharmacology, PI3K/Akt signaling pathway, NSCLC

## Abstract

**Background:**

Hedysarum Multijugum Maxim–Curcumae Rhizoma (HMMCR), a well-known herb pair in traditional Chinese medicine (TCM), has been widely used for the treatment of various cancers. However, the active components of HMMCR and the underlying mechanism of HMMCR for non-small-cell lung carcinoma (NSCLC) remain unclear.

**Methods:**

Active ingredients of HMMCR were detected by liquid chromatography electrospray ionization tandem mass spectrometry (LC-ESI-MS/MS). On this basis, potential targets of HMMCR were obtained from SwissTargetPrediction database. NSCLC-related targets were collected from four public databases (GeneCards, OMIM, TTD, and PharmGkb). The drug ingredients–disease targets network was visualized. The hub targets between HMMCR and NSCLC were further analyzed by protein–protein interaction (PPI), Gene Ontology (GO), and Kyoto Encyclopedia of Genes and Genomes (KEGG) pathway enrichment analyses. Subsequently, the results predicted by network pharmacology were further validated *via in vitro* experiments.

**Results:**

A total of 181 compounds were identified from the aqueous extract of HMMCR. Through network analysis, a compound–target network including 153 active ingredients of HMMCR and 756 HMMCR-NSCLC co-targets was conducted; 6 crucial compounds and 62 hub targets were further identified. The results of KEGG enrichment analysis showed that PI3K/Akt signaling pathway may be the critical pathway of HMMCR in the treatment of NSCLC. The *in vitro* experiments indicated that HMMCR inhibits the proliferation and migration of NSCLC cells *via* inactivation of the PI3K/Akt signaling pathway, consistent with the results predicted by network pharmacology.

**Conclusion:**

Integrating LC-ESI-MS/MS, network pharmacology approach, and *in vitro* experiments, this study shows that HMMCR has vital therapeutic effect on NSCLC through multi-compound, multi-target, and multi-pathway, which provides a rationale for using HMMCR for the treatment of NSCLC.

## Introduction

Lung cancer (LC), one of the most frequently diagnosed cancers, remains the leading cause of cancer-related deaths (1). According to the Global Cancer Statistics 2020, there were approximately 2.2 million new cases and 1.8 million deaths of lung cancer in 2020, which presents a major public health problem and an enormous burden on society worldwide (2, 3). In the past decades, with the development of early screening, molecular diagnosis, and multiple therapeutic techniques, the outcome of lung cancer patients has improved significantly (4–6). However, due to the high malignancy of lung cancer, the pathogenesis and metastasis mechanisms have not yet been fully elucidated. Most patients are already at an advanced stage when they are initially diagnosed. The prognosis of lung cancer patients remains poor. Non-small cell lung cancer (NSCLC) is the main pathological form of lung cancer, approximately accounting for about 85% of total lung cancer cases (7). Therefore, it is of great significance to further explore the mechanism of NSCLC and develop novel anti-lung cancer drugs.

In China, traditional Chinese medicine (TCM) preparations were widely used in cancer treatment and have unique advantages, especially in reducing the side effects of radiotherapy and chemotherapy, drug resistance, and prolonging the survival of cancer patients (8–10). According to the theory of TCM, supplementing Qi and activating blood circulation (SQ-ABC) is one of the important treatment methods for cancer. Hedysarum Multijugum Maxim–Curcumae Rhizoma (HMMCR) is a representative herb pair of SQ-ABC. Hedysarum Multijugum Maxim (HMM, called Huangqi in Chinese) is the dried root of *Astragalus membranaceus* (Fisch.) Bge (11). Curcumae Rhizoma (CR, called Ezhu in Chinese) is the dried rhizome of *Curcuma phaeocaulis* Valeton (12). Numerous studies have proven that HMMCR or its active components exert anti-cancer effects in various malignancies. Yong Bian et al. found that Huangqi and Ezhu decoction inhibits the proliferation and migration of colorectal cancer SW620 cells *via* inactivation of the Wnt5/β-catenin signaling pathway (13). Chengyong Xu et al. reported that extracts from HMMCR inhibit Lewis lung carcinoma cell growth in a xenograft mouse model by regulating mitogen-activated protein kinase (MAPK) signaling pathway, vascular endothelial growth factor (VEGF) production, and tumor angiogenesis (14). Kaempferol is an important component of HMM. Tae Woo Kim et al. showed that kaempferol activates the IRE1-JNK-CHOP signaling from the cytosol to the nucleus, and G9a inhibition activates autophagic cell death in gastric cancer cells (15). Curcumol, isolated from the CR, inhibits the malignant progression of prostate cancer PC3 cells and regulates the PDK1/AKT/mTOR pathway by targeting miR-9 (16). Moreover, *Astragalus* polysaccharide injection (a polysaccharide isolated from HMM) and β-elemene (a terpenoid of CR) have been widely used as new drug for the treatment of tumors in China (17, 18). However, the chemical compounds and mechanism of HMMCR to treat NSCLC have not been fully elucidated.

**Figure 1:**
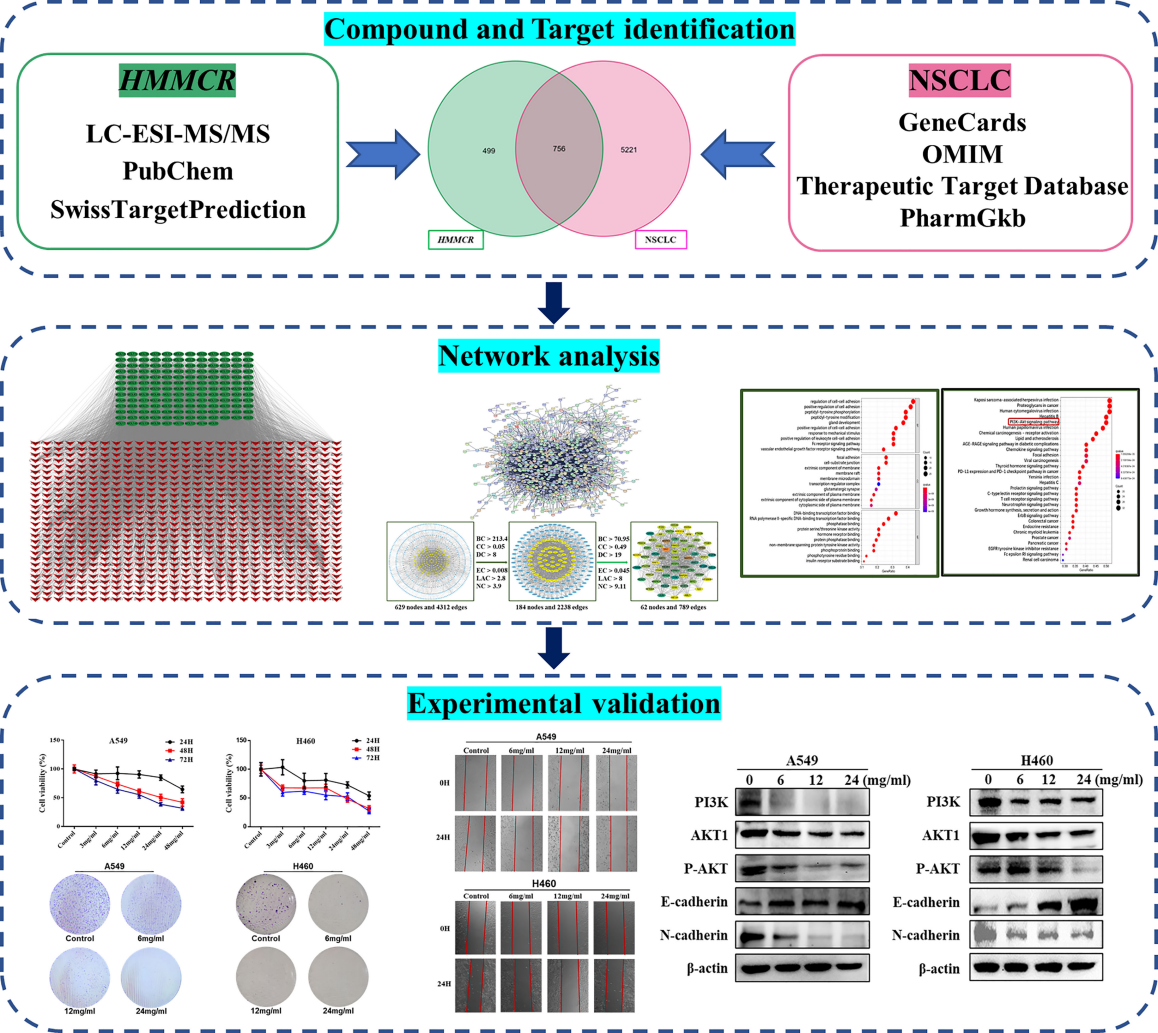
The framework and detailed idea of this study.

Network pharmacology, a novel comprehensive analysis tool based on large databases, can help researchers reveal the therapeutic mechanisms of TCM compounds by predicting the potential relationships between drugs, targets, and diseases (19). The purpose of this study was to detect the chemical components of HMMCR by liquid chromatography electrospray ionization tandem mass spectrometric (LC-ESI-MS/MS), predict the intervention pathways of HMMCR for NSCLC through network pharmacology, and further verify the underlying molecular mechanism through *in vitro* experiments. The framework and detailed idea of this study are shown in **Figure 1**.

## Materials and Methods

### Preparation of HMMCR Aqueous Extract

Two crude drugs of HMMCR were obtained from the Pharmacy Department of Dongfang Hospital, Beijing University of Chinese Medicine (Beijing, China). Hedysarum Multijugum Maxim (Huangqi, batch No. 20210412) were collected from the province of Neimenggu, China; Curcumae Rhizoma (Ezhu, batch No. 19062001) were collected from the province of Guangxi, China. The quality of each crude drugs was strictly ensured according to Chinese Pharmacopoeia to guarantee quality control (20). HMMCR aqueous extract was prepared according to the following experimental steps: 30 g of HMM and 15 g of CR were soaked in 10 volumes of distilled water for 30 min; then, the mixture was decocted for 1 h, and the resulting supernatant was collected. This extraction procedure was repeated twice, the supernatants obtained from two decoctions were mixed and evaporated to 45 ml, and the HMMCR with a concentration of 1 g/ml was prepared. Finally, the solution was passed through a filter with a 0.22-μm pore diameter and stored at −20°C.

### Compounds Detection and Targets Screening of HMMCR

Compounds of HMMCR were analyzed using liquid chromatography–mass spectrometry (LC-ESI-MS/MS) system (UHPLC, Thermo Fisher U3000 ultrahigh performance liquid chromatograph; MS, Thermo Scientific Q Exactive Plus™ Orbitrap MS system). The identification of unknown compound was performed by Compound discover 3.1, with mzcloud and mzVault databases. SwissTargetPrediction (http://www.swisstargetprediction.ch/), a public database for ligand-based target prediction of small biologically active molecule, was used to predict the potential targets of HMMCR. We transformed each compound identified by LC-ESI-MS/MS into canonical SMILES through the PubChem (https://pubchem.ncbi.nlm.nih.gov/). Subsequently, these SMILES were imported into SwissTargetPrediction to predict targets of compounds. Species were selected as “*Homo sapiens*” with probability >0 as the screening condition.

### HMMCR Targets Predicting and Compound Target Network Construction

The keyword “non-small cell lung cancer” was utilized to collect disease-related targets from the following databases: the GeneCards database (http://www.genecards.org/), Online Mendelian Inheritance in Man (OMIM, http://www.omim.org/) databases, Therapeutic Target Database (TTD, https://db.idrblab.org/ttd/), and PharmGkb (https://www.pharmgkb.org/). The overlapped targets among putative targets of the chemical compounds of HMMCR and NSCLC-related targets were considered as the potential targets of HMMCR for non-small cell lung cancer, which was visualized with a Venn diagram. Cytoscape 3.9.0 (https://cytoscape.org/) was applied to establish HMMCR active compounds–NSCLC targets network.

### PPI Network Construction and Hub Network Topological Screening

PPI network was constructed by the Search Tool for the Retrieval of Interacting Genes/Proteins (STRING; https://string-db.org/), using the overlapping targets among targets of HMMCR active ingredients and NSCLC-related targets, with the species limited to “*Homo sapiens*” and minimum required interaction score selected as a highest confidence score (0.900). The topological property of each node in the PPI network was evaluated by calculating six parameters with a Cytoscape plugin CytoNCA: “betweenness centrality (BC)”, “closeness centrality (CC)”, “degree centrality (DC)”, “eigenvector centrality (EC)”, “network centrality (NC)”, and “local average connectivity (LAC)”. Subsequently, nodes above twofold median values were selected as key targets and the hub nodes of HMMCR on NSCLC were constructed.

### Gene Ontology and KEGG Pathway Enrichment Analyses

Gene ontology (GO) functional annotation and Kyoto Encyclopedia of Genes and Genomes (KEGG) pathway enrichment were conducted in R (version: 3.6.3) using the “ClusterProfiler” package, and *p*. adjust (FDR) <0.05 was considered statistically significant.

### Reagents

The following reagents were used: Cell Counting Kit (CCK-8) assay solution and Cell Cycle Detection Kit (KeyGEN BioTECH, Beijing, China); Bicinchoninic Acid (BCA) Protein Assay Kit and bovine serum albumin (BSA), RIPA cell lysis buffer, and penicillin-streptomycin solution (Beyotime Institute of Biotechnology, Shanghai, China); Fetal bovine serum (FBS, Gibco, Grand Island, USA); SuperSignal Chemiluminescent HRP Substrate (Thermo Fisher scientific Inc., Rockford, USA); Trypsin-EDTA (Invitrogen, Rockville, USA); Dulbecco’s minimum essential medium (DMEM, Corning Incorporated, New York, USA); antibodies against AKT1, p-AKT, PI3K, E-cadherin, N-cadherin, and β-actin (ProteinTech Group, Chicago, USA).

### Cell Line and Culture

Human NSCLC cell lines NCI-A549 and NCI-H460 were kindly gifted by Professor Zongfang Zheng from Peking University Health Science Center. Cells were cultured in DMEM medium supplemented with 10% fetal bovine serum (FBS) and 100 U/ml of penicillin–streptomycin solution and maintained at 37°C in a humidified incubator with 5% CO_2_.

### Cell Viability Assay

Cell viability was explored using CCK-8 solution. Briefly, A549 cells and H460 cells (5×10^3^ cells/100μl/well) in 96-well plates were exposed to HMMCR at different concentrations (0, 3, 6, 12, 24, or 48 mg/ml) for 24, 48, and 72 h. Ten microliters of CCK-8 reagent was added to each well, and cells were incubated for 2 h at 37°C and 5% CO_2_. A microplate reader was used to measure the optical density (OD) at 450 nm of each well.

### Colony Formation Evaluation

Cells (2,000 cells/well) in six-well plate were cultured overnight before being exposed to different treatment conditions for 48 h and cultured in complete medium for additional 12 days. Colonies were fixed for 15 min at room temperature in methanol and stained for 15 min in crystal violet.

### Wound Healing Assay

Wound healing assay was used to determine the migration of A549 and H460 cells. Briefly, a straight line was marked using a marker pen on the back of the well before cells (4×10^5^ cells/2 ml/well) were seeded in six-well plates. After cells were grown to 90% confluence, the cell was scraped lightly with the 200-μl tip of the spear to draw another straight line, perpendicular to the marking line on the back of each well at the center. Subsequently, cells were then rinsed with sterile phosphate-buffered saline (PBS) for three times and cultured with serum-free DMEM medium. The wound healing was observed and photographed at the time point of 0 and 24 h with different concentrations (0, 6, 12, or 24 mg/ml) of HMMCR. Image J Software was used to calculate the migrated distance.

### Western Blot Investigation

Total protein was extracted from A549 and H460 cells challenged with different concentrations (0, 6, 12, or 24 mg/ml) of HMMCR for 48 h. RIPA Cell Lysis Buffer was used to lyse cells on ice for 30 min, followed by centrifuged at 15,000 *g* at 4°C for 20 min. Bicinchoninic acid (BCA) method was used to detect the protein concentration. Proteins (25 μg/sample) were separated by sodium dodecyl sulfate–polyacrylamide gel electrophoresis (SDS-PAGE) and transferred to polyvinylidene fluoride (PVDF) membranes. After blocking in 5% BSA for 1 h at room temperature, the membranes were blotted with primary and secondary antibodies, respectively, then detected by the Enhanced Chemiluminescence Detection Kit. ChemiDoc MP Imaging System and the software Image Lab version were applied to acquire images of the Western blot. Image J Software was used to calculate the grayscale value.

### Statistical Analysis

All results shown in the current study are expressed as mean ± standard deviation (SD). SPSS 21.0 software was used for statistical analysis. *t*-Test was used for differences between the two groups. Differences between multi-groups were assessed by one-way ANOVA. *p < 0.05, **p < 0.01, and ***p < 0.001 indicated statistically significant difference.

## Results

### Active Compounds and Putative Targets of HMMCR

HMMCR aquatic extract samples were analyzed by LC-ESI-MS/MS. A total of 181 compounds of HMMCR were detected (**Supplementary Table S1**). The secondary mass spectra of each compound are shown in **Supplementary Table S2**. SwissTargetPrediction was used to predict the targets of each compounds. Twenty-eight components (MOL3, MOL7, MOL42, MOL47, MOL49, MOL57, MOL63, MOL68, MOL74, MOL81, MOL84, MOL92, MOL95, MOL98, MOL99, MOL109, MOL114, MOL115, MOL141, MOL156, MOL157, MOL158, MOL161, MOL164, MOL165, MOL166, MOL170, and MOL178) without canonical SMILES or targets were eliminated. SwissTargetPrediction predicted a total of 13,896 potential targets of the rest of 153 compounds, and we obtained 1,255 after removing duplicate targets (**Supplementary Table S3**).

### NSCLC Targets Prediction and Network Construction

**Figure 2:**
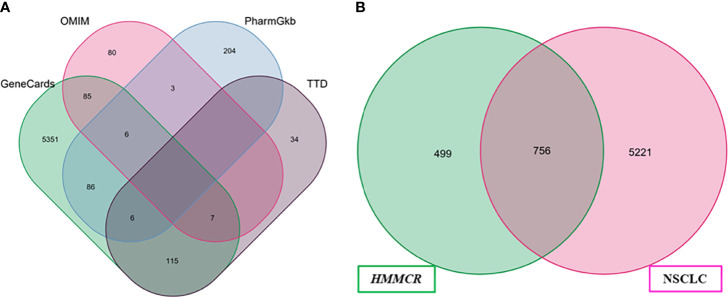
**(A)** The Venn diagram of NSCLC-related targets obtained from four public database. **(B)** Venn diagram of compounds and NSCLC targets.

**Figure 3:**
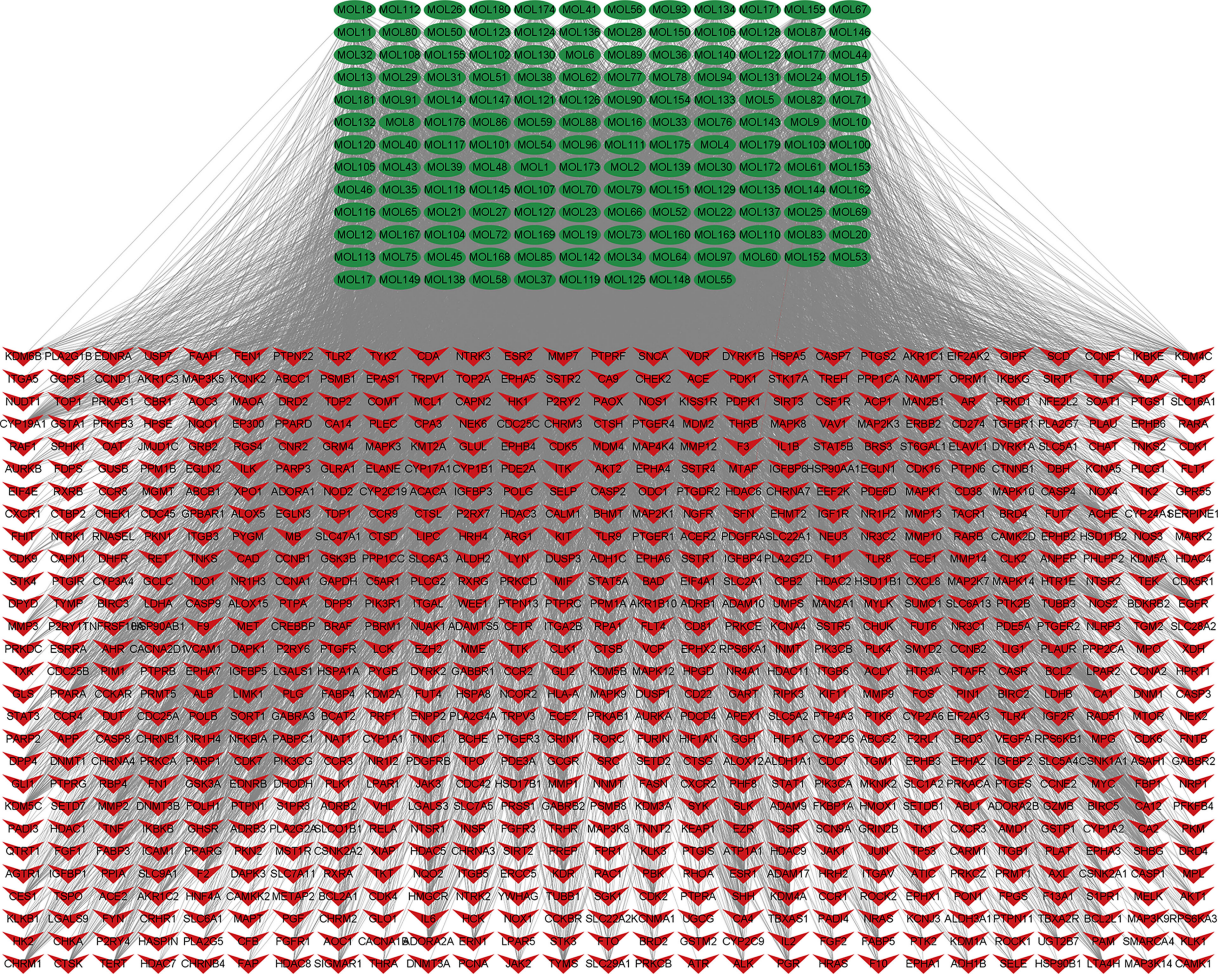
The HMMCR active compounds–NSCLC targets network. The green circle represents compounds present in HMMCR, and the red circle represents targets.

A total of 5,977 NSCLC-related targets were identified after comprehensive searching from GeneCards, OMIM, TTD, and PharmGkb databases (**Supplementary Table S4**), and the Venn diagram of NSCLC targets is shown in **Figure 2A**. Furthermore, a Venn plot showing the intersections of HMMCR and NSCLC-related targets is shown in **Figure 2B**. The result shows that a total of 756 targets of HMMCR were overlapped with those of non-small cell lung cancer, which indicated that HMMCR has a strong therapeutic effect on NSCLC, and its anti-cancer mechanism is worthy of further exploration. In addition, the HMMCR active compounds–NSCLC targets network was further constructed, which included 909 nodes and 9,186 edges, indicating that HMMCR exerts anti-NSCLC effects through multiple ingredients and targets (**Figure 3**). The green circle represents the ingredients of HMMCR, and the red triangle represents 756 targets. Six key compounds were identified with degree ≥80, namely, hispidulin (MOL155), 5-hydroxy-6,7-dimethoxylflavone (MOL113), genistein (MOL108), (15Z)-9,12,13-trihydroxy-15-octadecenoic acid (MOL14), isokaempferide (MOL55), and naringenin (MOL106). The top 20 components by degree are shown in **Table 1**.

**Table 1:** The top 20 components of HMMCR by degree.

ID	Compounds	Degree
MOL155	Hispidulin	84
MOL113	5-Hydroxy-6,7-dimethoxylflavone	83
MOL108	Genistein	81
MOL14	(15Z)-9,12,13-Trihydroxy-15-octadecenoic acid	80
MOL55	Isokaempferide	80
MOL106	Naringenin	80
MOL2	Calycosin	79
MOL101	Liquiritigenin	79
MOL129	Lariciresinol 4-O-glucoside	79
MOL142	Sinapinic acid	79
MOL119	4-Coumaric acid	78
MOL126	Daidzein	78
MOL127	Isoferulic acid	78
MOL179	Shogaol	78
MOL103	Biochanin A	77
MOL125	Ageratriol	77
MOL174	Wilforlide A	77
MOL5	Formononetin	76
MOL78	Isosakuranetin	76
MOL107	Ferulaldehyde	75

### PPI Network and Hub Targets Analyses

**Figure 4:**
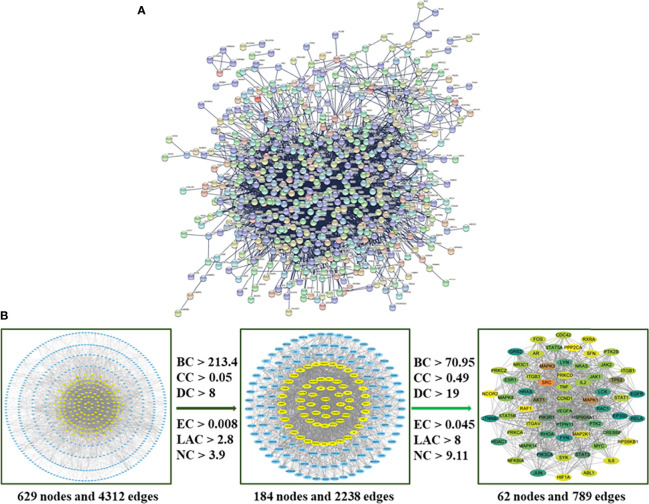
**(A)** The PPI network of 756 intersection targets from the HMMCR for treatment of NSCLC. **(B)** The process of hub network topological screening. The BC, CC, DC, EC, LAC, and NC values of each target are calculated; nodes higher than twice the median value are further selected as hub targets.

The 756 compound–disease co-targets were uploaded to the STRING database. A PPI network contains 629 nodes, and 4,312 edges were established, which indicated that there are high connectivity relationships between these targets (**Figure 4A**). To further explore the hub targets of HMMCR on NSCLC, topological analysis was performed on the PPI network according to six parameters of “BC”, “CC”, “DC”, “EC”, “NC”, and “LAC” and select nodes above twofold median values as hub targets. The threshold values of the first screening were BC > 213.4, CC > 0.05, DC > 8, EC > 0.008, LAC > 2.8, and NC > 3.9, and the results were 184 hub nodes and 2,238 edges. Subsequently, the second screening threshold values were BC > 70.95, CC > 0.49, DC > 19, EC > 0.045, LAC > 8, and NC > 9.11. The results were 62 hub nodes and 789 edges (**Figure 4B**). Therefore, the results of hub targets network indicated that 62 hub targets may account for the crucial therapeutic effects of HMMCR on NSCLC (**Table 2**).

**Table 2:** Sixty-two hub targets of HMMCR for NSCLC.

Number	Target	Degree	Number	Target	Degree	Number	Target	Degree
1	SRC	48	22	JUN	28	43	MAP2K1	21
2	MAPK1	45	23	JAK1	27	44	CDC42	20
3	MAPK3	44	24	ESR1	27	45	HDAC1	20
4	STAT3	40	25	STAT5A	27	46	PRKCA	19
5	LCK	37	26	TP53	26	47	PRKCZ	19
6	PIK3CA	37	27	EP300	26	48	AR	19
7	PIK3R1	37	28	CTNNB1	26	49	CCND1	19
8	EGFR	35	29	STAT1	26	50	RPS6KB1	18
9	FYN	34	30	CREBBP	24	51	HIF1A	18
10	AKT1	33	31	NRAS	24	52	RAF1	17
11	HSP90AA1	32	32	STAT5B	24	53	ITGAV	17
12	RAC1	31	33	FOS	24	54	SFN	16
13	PTPN11	31	34	MYC	24	55	ITGB1	16
14	GRB2	31	35	MAPK8	24	56	ITGB3	16
15	LYN	31	36	VEGFA	24	57	ABL1	16
16	RELA	30	37	PTK2B	23	58	PPP2CA	16
17	MAPK14	30	38	SYK	23	59	IL6	15
18	IL2	29	39	JAK2	23	60	TNF	15
19	RHOA	29	40	NR3C1	22	61	RXRA	15
20	HRAS	28	41	NFKBIA	21	62	NCOR2	12
21	PTK2	28	42	PRKCD	21			

### GO and KEGG Pathway Enrichment Analysis

**Figure 5:**
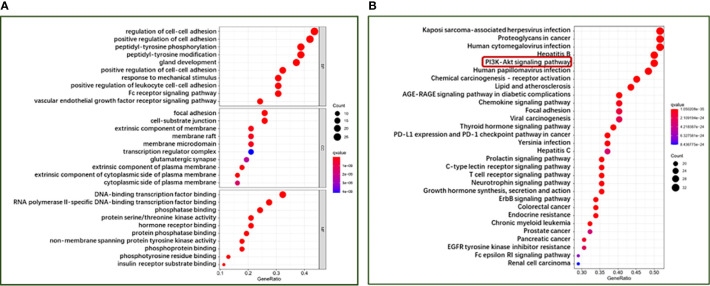
The bubble diagram of **(A)** GO function enrichment and **(B)** KEGG pathway enrichment analysis of 62 hub targets from the HMMCR for treatment of NSCLC. The larger the number of enriched targets, the bigger the dots; the smaller the p-value, the redder the color of the dot.

**Figure 6:**
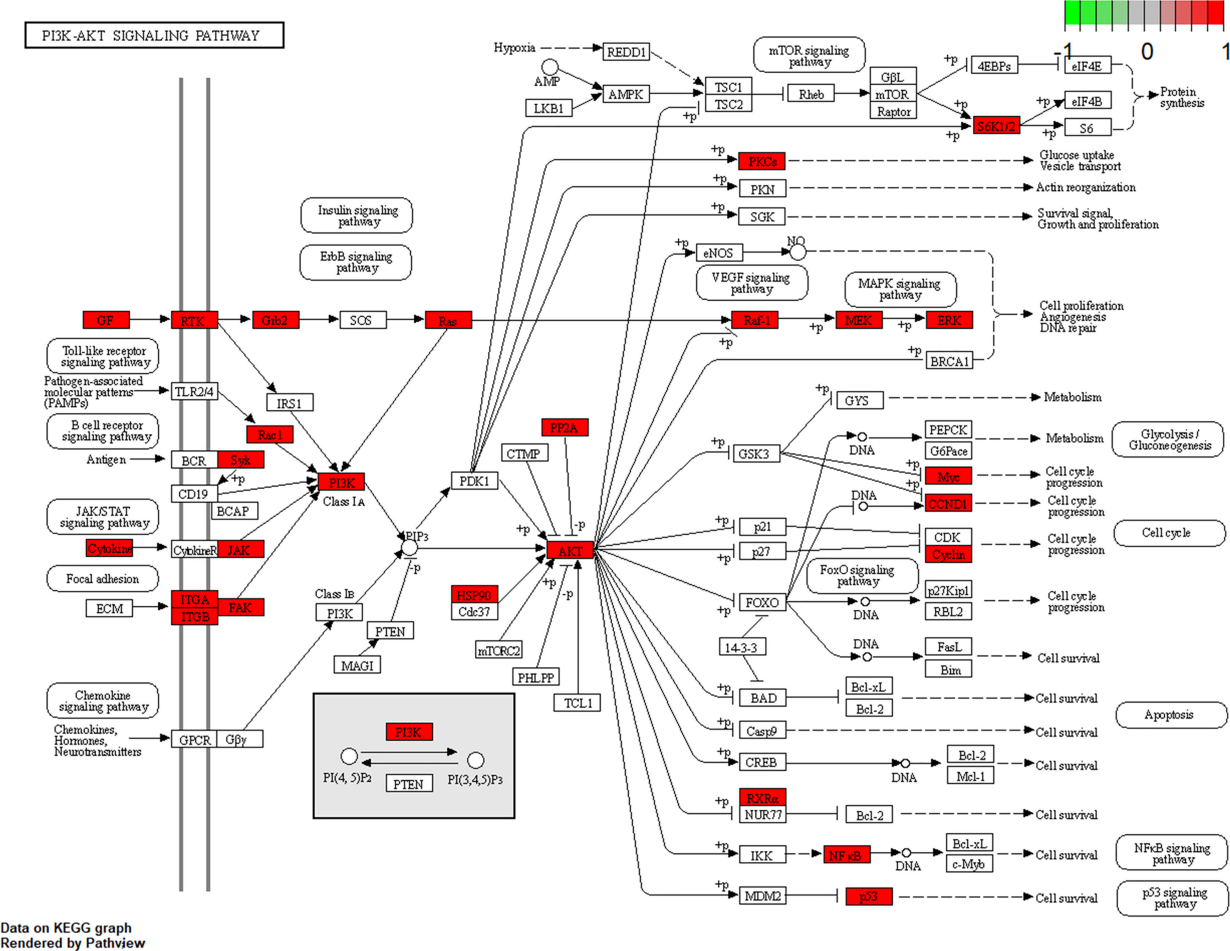
The maps of PI3K/AKT signaling pathway. The white rectangles and red rectangles represent unidentified proteins and identified proteins, respectively.

To further explore the potential therapeutic mechanisms of HMMCR on NSCLC, the 62 hub targets were performed by GO and KEGG pathway enrichment analyses. A total of 2,150 biological process (BP), 101 cellular component (CC), and 171 molecular function (MF) terms were enriched (**Supplementary Table S5**). The top 10 strongly enriched GO terms in BP, CC, and MF are shown in **Figure 5A**. The results showed that the terms of BP mainly contained regulation of cell–cell adhesion, positive regulation of cell adhesion, and peptidyl-tyrosine phosphorylation; the terms of CC mainly contained focal adhesion, cell–substrate junction, and extrinsic component of membrane; the terms of MF mainly contained DNA-binding transcription factor binding, RNA polymerase II-specific DNA-binding transcription factor binding, and phosphatase binding. Furthermore, 172 pathways were enriched (**Supplementary Table S5**). The top 30 significantly enriched pathways of HMMCR on NSCLC are shown in **Figure 5B**. The results indicated that PI3K/Akt signaling pathway may be the critical pathway of HMMCR in the treatment of NSCLC (**Figure 6**).

### HMMCR Inhibited the Proliferation of NSCLC Cells

**Figure 7:**
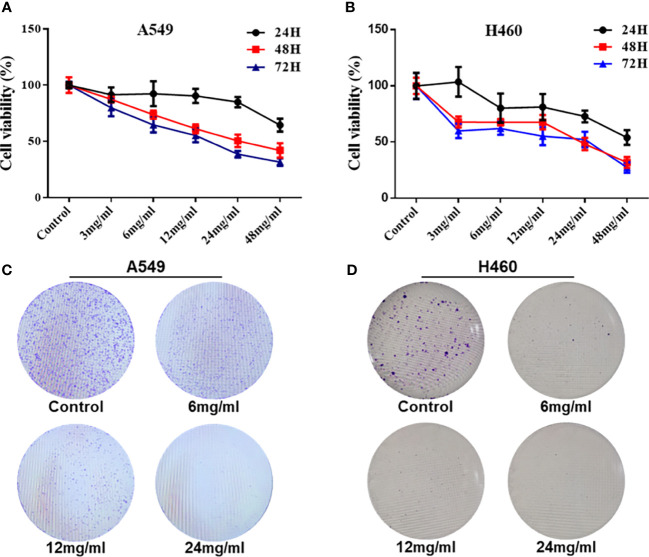
HMMCR inhibits the proliferation of NSCLC cells. The viabilities of **(A)** A549 and **(B)** H460 cells after HMMCR treatment at the doses of 0–48 mg/ml for 0–72 h were measured by CCK-8. The self-renew ability of **(C)** A549 and **(D)** H460 cells after HMMCR treatment at the doses of 0–24 mg/ml for 48 h were measured by colony formation assay.

To explore the effect of HMMCR on NSCLC cell proliferation, viable cell numbers were continuously detected by CCK-8 for 72 h when A549 and H460 cells were challenged with different concentrations of HMMCR (0, 3, 6, 12, 24, or 48 mg/ml). Results showed that HMMCR dose- and time-dependently inhibited the viabilities of A549 and H460 cells (**Figures 7A, B** **)**. Meanwhile, the result of colony formation assay demonstrated that HMMCR significantly reduced the colony formation of A549 and H460 cells (**Figures 7C, D** **)**. These above results indicated that HMMCR can significantly inhibit the proliferation of NSCLC cells *in vitro*.

### HMMCR Suppressed the Migration of NSCLC Cells

**Figure 8:**
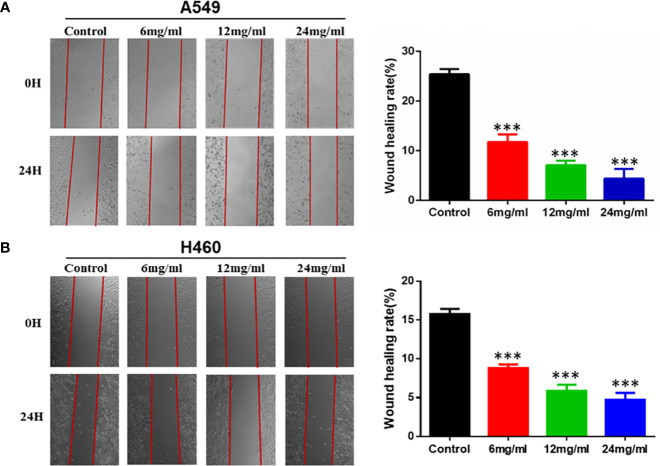
HMMCR inhibits the migration of NSCLC cells. The migration of **(A)** A549 and **(B)** H460 cells after HMMCR treatment at the doses of 0–24 mg/ml for 24 h were determined by wound healing assay and quantified by Image J software. ***p < 0.001 vs. control.

To further investigate the effect of HMMCR on the NSCLC cells migratory ability, A549 and H460 cells were treated for 24 h with 0, 6, 12, or 24 mg/ml of HMMCR and the effect determined using wound-healing migration assay. Our results indicated that migration of A549 and H460 cells was inhibited by HMMCR in a dose-dependent manner (**Figure 8**). Therefore, the results indicated that HMMCR can significantly inhibit the migration of NSCLC cells *in vitro*.

### HMMCR Inhibited the PI3K/Akt Pathway in NSCLC Cells

**Figure 9:**
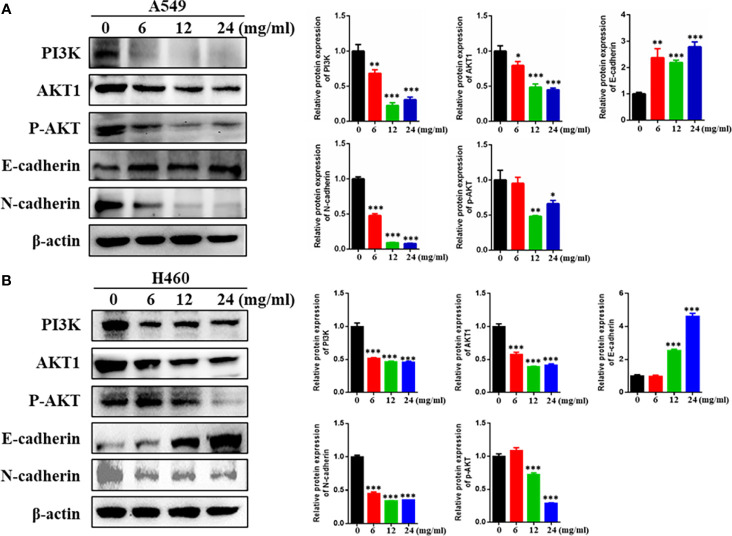
HMMCR suppresses NSCLC cells progression *via* targeting PI3K/Akt pathway. PI3K, AKT1, p-AKT, E-cadherin, and N-cadherin expressions in **(A)** A549 and **(B)** H460 cells after HMMCR treatment at the doses of 0–24 mg/ml for 48 h were determined by Western blot assay. β-Actin was used as an internal control. All data were quantified by Image J software. *p < 0.05, **p < 0.01, ***p < 0.001 vs. control.

To verify the molecular mechanism of HMMCR for NSCLC, the key protein of PI3K/Akt pathway that predicted by network pharmacological analysis was detected by Western blotting. As shown in **Figure 9**, our results showed that PI3K, AKT1, and p-AKT protein levels were significantly reduced in a dose-dependent manner after treatment of A549 and H460 cells with HMMCR. Meanwhile, cell-migratory-related protein (E-cadherin and N-cadherin) were further analyzed. Our data indicated that expression of N-cadherin was decreased and E-cadherin was increased in a dose-dependent manner. The results demonstrated that HMMCR inhibited the proliferation and migration of NSCLC cells at least through the PI3K/Akt pathway, thereby achieving an anti-cancer effect.

## Discussion

The occurrence and development of non-small cell lung cancer is an extremely complex and multifaceted biological process (21). Exploring the mechanism of NSCLC and developing new anti-tumor drugs are still important research directions in the field of oncology. TCM believes that “Qi deficiency and blood stasis (QD-BS)” is the key pathogenesis of lung cancer. QD-BS causes poor blood circulation in tumors, which is similar to the hypercoagulable state of tumor patients found in modern medicine. The formation of tumor thrombi is closely related to abnormal coagulation during tumor progression (22). Due to the characteristics of promoting blood circulation and improving the hypoxic microenvironment, a variety of Chinese herb and formulas for SQ-ABC have become vital adjuvant treatments in treatment of tumor, such as HMMCR, Huayu pill (23, 24), Taohong Siwu Decoction (25), and Qizhu decoction (26).

HMMCR, a typical representative herb pair of SQ-ABC, has been used in treating various cancer for many years. In recent years, the effective ingredients and the anti-cancer mechanism of HMMCR attracted the attention of numerous researchers. Qian Wang et al. verified seven components of HMMCR by HPLC, including Calycosin, Formononetin, Curcumenol, Astragaloside A, Astragaloside I, Calycosin-7-glucoside, and Astragaloside II. Meanwhile, HMMCR can significantly inhibit tumor growth, without toxifying the liver and kidney (27). Chengyong Xu et al. suggested that *Astragalus* polysaccharide and *Curcumin* were the optimal combination of HMM and CR, which could initiate apoptosis of A549 cells under chemical-induced hypoxia *via* increasing the expression of Bax and caspase-3, decreasing the expression of Bcl-2 (28). However, due to the complexity of the ingredients, the compounds and mechanism of HMMCR to treat NSCLC have not been fully elucidated. In this study, we applied LC-ESI-MS/MS to detect the active compounds of HMMCR and further used network pharmacology approach to predict the pharmacological mechanism of HMMCR on NSCLC, following validation by *in vitro* experiments.

Based on the results of LC-ESI-MS/MS, a total of 181 compounds of HMMCR were identified. One hundred fifty-three compounds and 1,255 potential targets of HMMCR were included after screening by SwissTargetPrediction. In addition, 5,977 NSCLC-related targets were identified after comprehensively searching from GeneCards, OMIM, TTD, and PharmGkb databases. In a comparison of the 1,255 targets of HMMCR with the 5,977 NSCLC-related targets, 756 targets of HMMCR were overlapped, which indicated that HMMCR has a strong therapeutic effect on non-small cell lung cancer, and its anti-cancer mechanism is worthy of further exploration. Furthermore, six key compounds were identified with degree ≥80, namely, hispidulin, 5-Hydroxy-6,7-dimethoxylflavone, genistein, (15Z)-9,12,13-Trihydroxy-15-octadecenoic acid, isokaempferide, and naringenin. The results of hub targets network indicated that 62 hub targets (SRC, MAPK1, MAPK3, STAT3, LCK, PIK3CA, PIK3R1, EGFR, FYN, AKT1, HSP90AA1, RAC1, PTPN11, GRB2, LYN, RELA, MAPK14, IL2, RHOA, HRAS, PTK2, JUN, JAK1, ESR1, STAT5A, TP53, EP300, CTNNB1, STAT1, CREBBP, NRAS, STAT5B, FOS, MYC, MAPK8, VEGFA, PTK2B, SYK, JAK2, NR3C1, NFKBIA, PRKCD, MAP2K1, CDC42, HDAC1, PRKCA, PRKCZ, AR, CCND1, RPS6KB1, HIF1A, RAF1, ITGAV, SFN, ITGB1, ITGB3, ABL1, PPP2CA, IL6, TNF, RXRA, and NCOR2) might be the most important targets of HMMCR for NSCLC. GO enrichment analysis confirmed the biological functions of the 62 hub targets of HMMCR against NSCLC. Furthermore, the results of KEGG pathway enrichment analysis showed that 172 related signaling pathways were obtained; PI3K/Akt signaling pathway may be the critical pathway of HMMCR in the treatment of NSCLC.

Furthermore, *in vitro* experiments were performed to validate the results of network pharmacology. Our results indicated that HMMCR inhibited the viability and migration of NSCLC A549 and H460 cells. Meanwhile, we further detected the key protein expression of PI3K/Akt pathway. Increasing studies demonstrated that PI3K/Akt pathway was involved in the occurrence and development of various malignancies, including colorectal cancer, breast cancer, gastric cancer, and ovarian cancer (29–32). Briefly, the cytokines and growth factors can activate PI3K, followed by promoting Akt phosphorylation and regulating cell proliferation, thereby promoting tumor growth (33). Our results further demonstrated that HMMCR inhibited PI3K, AKT1, and p-AKT protein expression levels in a dose-dependent manner. Therefore, HMMCR can inhibit the proliferation and migration of lung cancer A549 and H460 cells through regulating the PI3K/Akt pathway, which further support the network pharmacological prediction.

Taken together, this is the first time that LC-ESI-MS/MS is used to detect the active compounds of HMMCR and integrate network pharmacology and *in vitro* experiments to explore the pharmacological mechanisms of HMMCR for non-small cell lung cancer, suggesting that HMMCR can efficiently inhibit NSCLC cellular proliferation and migration by regulating the PI3K/Akt signaling pathways. However, several limitations in this study must be emphasized. First, *in vivo* experiments and more cell lines are needed to further validate the effect and mechanism of HMMCR for NSCLC. Moreover, other signaling pathways (e.g., PD-L1 expression and PD-1 checkpoint pathway in cancer, Proteoglycans in cancer, and JAK-STAT signaling pathway) predicted by network pharmacology might also be involved in the anti-NSCLC effect of HMMCR, which needs further verification. Despite these limitations, our study provides powerful theoretical basis for using HMMCR in the treatment of NSCLC.

## Conclusion

In summary, LC-ESI-MS/MS, network pharmacology and *in vitro* experiments validation were utilized to explore the active ingredients of HMMCR and their mechanisms for non-small cell lung cancer. The results of our study demonstrated that HMMCR might inhibit the cell proliferation and reduce the migration of NSCLC cells, mainly *via* regulating PI3K/Akt signaling pathways. This study provides a rationale for using HMMCR in the treatment of NSCLC.

## Data Availability Statement

The datasets presented in this study can be found in online repositories. The names of the repository/repositories and accession number(s) can be found in the article/**Supplementary Material**.

## Author Contributions

Conception and design: SH, KH, and LG. Experimental operation: SH. Manuscript writing and data analysis: SH, MG, and SZ. Manuscript revision: MJ, KH, and LG. All authors contributed to the article and approved the submitted version.

## Funding

This study was supported by grants from National Natural Science Foundation of China (No. 82174458), the Fundamental Research Funds for the Central Universities (2021-JYB-XJSJJ-066, 2020-JYB-ZDGG-123), and New Innovation Project-Yiqilin Leading Talent Project.

## Conflict of Interest

The authors declare that the research was conducted in the absence of any commercial or financial relationships that could be construed as a potential conflict of interest.

## Publisher’s Note

All claims expressed in this article are solely those of the authors and do not necessarily represent those of their affiliated organizations, or those of the publisher, the editors and the reviewers. Any product that may be evaluated in this article, or claim that may be made by its manufacturer, is not guaranteed or endorsed by the publisher.
